# Training infectious diseases fellows for a new era of hospital epidemiology

**DOI:** 10.1017/ash.2021.186

**Published:** 2021-10-06

**Authors:** Elise M. Martin, Graham M. Snyder

**Affiliations:** 1 Department of Infection Prevention and Control, UPMC Presbyterian, Pittsburgh, Pennsylvania; 2 Division of Infectious Diseases, Department of Medicine, University of Pittsburgh School of Medicine, Pittsburgh, Pennsylvania

## Abstract

Training programs for infectious diseases fellows pursuing a career in infection prevention and control and hospital epidemiology are grounded in mentorship and organizational experience. In this commentary, we propose a competency-based framework for creating structured learning for infectious diseases fellows pursuing hospital epidemiology and related fields.

## Evolving training to match a growing field

In recent decades, the field of infection prevention and control and healthcare epidemiology (IP&C/HE) has expanded considerably.^1^ IP&C/HE programs are not only responsible for surveillance and prevention of healthcare-associated infections (HAIs), they also conduct cluster investigations, contribute to emergency preparedness, engage in quality improvement efforts, and partner with patient safety, occupational health, and environmental health and safety teams. They employ diagnostic and antimicrobial stewardship strategies, and they develop both patient and staff education.^2^ The coronavirus disease 2019 (COVID-19) pandemic has only further highlighted the important roles of IP&C/HE and healthcare epidemiologists.^3,4^

As the field of IP&C/HE evolves in an increasingly complex healthcare environment, more demands are made on the healthcare epidemiologists who lead these programs.^5^ When IP&C programs were first developed, the physician team members made contributions from different backgrounds, including infectious diseases (ID), pathology, and microbiology, and not all had formal training in hospital epidemiology. These programs also varied in the amount of time dedicated.^5–7^ Although there is no formal accreditation process for healthcare epidemiologists, more ID physicians are seeking specific fellowship training in IP&C with the goal of a career in infection prevention as a healthcare epidemiologist, and healthcare facilities are offering positions with protected time to lead IP&C programs.

## Existing training resources

There is no Accreditation Council for Graduate Medical Education (ACGME) fellowship, other formal accreditation, or certification process for training to be a healthcare epidemiologist. The ACGME requires all ID fellows to have some training in infection prevention, including competence in the diagnosis and management of HAI and device-associated infections, as well as infections, as well as general knowledge of infection control and hospital epidemiology.^8^

Other educational opportunities include the Society for Healthcare Epidemiology of America (SHEA)/Centers for Disease Control and Prevention (CDC) Training Certification in Healthcare Epidemiology,^9^ the SHEA Primer on Healthcare Epidemiology, Infection Control, and Antimicrobial Stewardship (online ID fellows’ course),^10^ and the Annual Fellows’ Course in Healthcare Epidemiology, Infection Prevention, and Antimicrobial Stewardship (in-person course).^11^

Most ID fellows interested in a career in infection prevention seek out ID fellowship programs comprising an experienced hospital epidemiologist mentor and a strong IP&C program with a history of training ID fellows who have successfully launched careers as healthcare epidemiologists. Some of these programs have defined tracks in IP&C and may offer a third year of fellowship dedicated to additional IP&C training and research. The last SHEA membership survey published in 2010 found that while nearly 60% of respondents reported completing the SHEA/CDC training course, only 26% completed at least 1 year of dedicated IP&C training.^5^

At a series of national meetings, ID division chiefs and program directors recommended either a 1-month rotation in IP&C and/or course involving a combination of didactic and/or practical IP work.^12^ They did not define what should be included in the rotation or the didactics. Subsequent surveys of both adult and pediatric ID fellows and recent graduates highlighted the importance but shortcomings of IP&C training during fellowship. Only half of adult ID fellowship respondents thought IP&C training was adequate, and pediatric ID fellowships were reported to be deficient in both the breadth and depth of IP&C training in most programs.^13,14^ In at least one institution’s published experience, a month-long semistructured rotation can be successful in improving IP&C education among ID fellows.^15^

## A proposed structured training model

To standardize IP&C education for ID fellows at our institution and to ensure that key competencies are met, we developed a training model enumerating competencies for ID fellows entering IP&C/HE fields (ie, “track” competencies) and non-IP&C/HE fields (ie, nested “rotation” competencies), with concrete activities to achieve each (Table 1). The competencies were adapted in part from a published SHEA white paper outlining healthcare epidemiologist skills and competencies, as well as our professional medical education experience.^2^

**Table 1.** Infectious Diseases Fellowship Infection Prevention & Control/Hospital Epidemiology Rotation and Track Core Competencies and Activities

Competency	Activities	Rotation	Track
Surveillance and Reporting			
Understand how healthcare-associated infection (HAI) surveillance is conducted	Observe infection preventionists (IP) reviewing microbiology results and applying NHSN definitions for the following: Central-line–associated bloodstream infection (CLABSI) Catheter-associated urinary tract infection (CAUTI) Surgical site infection (SSI) Ventilator-associated event (VAE) *Clostridioides* (*Clostridium*) *difficile* and multidrug-resistant organisms	✓	✓
Consider methods of validation and auditing of HAI data	Discuss the internal validation of HAI data Identify the opportunities for external validation of HAI data		✓ ✓
Review the advantages and limitations of surveillance software in the process of HAI surveillance	Review with IP data analyst or informatics team members the sources of surveillance data Review the SHEA research methods white paper on administrative and surveillance data		✓ ✓
Distinguish between HAI surveillance and clinical definitions for infectious syndromes	Participate in a multidisciplinary discussion, quality review, or teaching session that includes case-based review describing surveillance-identified HAI	✓	✓
Understand and effectively utilize various HAI measures including numerator and denominator, and counts, rates, and adjusted rates	As part of routine IP work or project work, present HAI outcome data with appropriate case ascertainment, at-risk population, and consideration of risk adjustment	✓	✓
Evaluate facility performance on HAI and HAI prevention process measures using internal and external benchmarks	For at least 1 HAI, consider and discuss internal and external benchmarking options	✓	✓
Consider the role of HAI in the context of other patient safety events, including falls, pressure ulcers, and other adverse outcomes	Attend ≥1 patient safety or hospital quality meeting, and compare and contrast infection-related and non–infection-related outcomes including reporting, benchmarking, and improvement interventions		✓
Review requirements for reporting community- and healthcare-associated infections to public health	Review state and local health department reporting requirements Observe IP processes for identifying reportable illnesses and performing reporting	✓ ✓	✓ ✓
**Cluster detection, investigation, and resolution**			
Describe what defines an epidemiologically significant cluster, including differences in cluster detection among pathogens	Participate in the investigation and response to a single case of an epidemiologically significant pathogen, such as vancomycin nonsusceptible *Staphylococcus aureus*, carbapenem-resistant Enterobacteriaceae, *Candida auris*, *Legionella*, invasive mold, or SARS-CoV-2 (If no case is available, describe pathogens for which 1 or a few cases define an outbreak and those for whom clusters are differentiated from endemic rates)	✓	✓
For a multiple-case cluster, understand the application of multiple steps of an outbreak investigation	Participate in the investigation of a HAI cluster (If no cluster is available, describe from the published literature at least 1 description of an outbreak investigation)	✓	✓
Understand the roles of case–control or cohort study in identifying potential transmission routes leading to a cluster of infections	Perform a case-control or cohort study as part of a cluster investigation Describe the findings of a cluster investigation to stakeholders and hospital leadership, including proposed or enacted changes to baseline prevention practices		✓ ✓
Describe the role that identification of genetic relatedness plays in cluster identification, and understand commonly used techniques from antimicrobial phenotype to molecular typing and whole-genome sequencing (WGS)	Participate in a meeting where the use of WGS is considered for a possible cluster and/or results are reviewed Review ≥2 published articles of healthcare-associated outbreaks and be able to describe the methods used to establish genetic relatedness	✓ ✓	✓ ✓
Understand the key elements of an exposure investigation: identifying patients and providers potentially exposed; incubation period; postexposure measures including prophylaxis, vaccination, monitoring, furlough	Assist in an exposure investigation (eg, tuberculosis, varicella zoster, SARS-CoV-2) in conjunction with the infection preventionist		✓
Consider the role of active surveillance testing in the prevention of multidrug-resistant organisms	Compare published observational or clinical trial with institutional practice for ≥1 multidrug-resistant pathogen such as methicillin-resistant *S. aureus*, vancomycin-resistant enterococci, or carbapenem-resistant Enterobacteriaceae	✓	✓
**Pathogen transmission and transmission interruption**			
Understand modes of pathogen transmission	Be able to describe the common and potential modes of transmission of healthcare-associated pathogens	✓	✓
Describe the rationale for transmission-based precautions for pathogens commonly observed in the healthcare setting	Review the CDC guideline “Type and Duration of Precautions Recommended for Selected Infections and Conditions” and compare these to institutional practices	✓	✓
Identify clinical situations for which standard precautions (and specific elements of standard precautions) should be employed	Perform with infection preventionists observations of personal protective equipment use	✓	✓
Hand hygiene: understand the evidence base for hand hygiene as an infection prevention tool to reduce transmission in the healthcare setting	Review the World Health Organization guidelines on hand hygiene, and describe at least 1 high-quality study of the effectiveness of hand hygiene	✓	✓
Hand hygiene: observe the measurement and feedback of hand hygiene adherence	Participate in hand hygiene observations with a member of the IP team Conduct independent observations of hand hygiene adherence and provide feedback to observed units	✓	✓ ✓
Hand hygiene: understand interventions that may be employed to improve hand hygiene adherence in the acute care setting	Plan a unit-based intervention to improve hand hygiene adherence		✓
Be able to describe interventions to prevent catheter-related bloodstream infections	Compare and contrast institutional policies with evidence-based practices in SHEA Compendium of Strategies to prevent central-line–associated bloodstream infections	✓	✓
Be able to describe interventions to prevent catheter-related urinary tract infections	Compare and contrast institutional policies with evidence-based practices in SHEA Compendium of Strategies to prevent catheter-associated urinary tract infections	✓	✓
Be able to describe interventions to prevent ventilator-associated events	Compare and contrast institutional policies with evidence-based practices in SHEA Compendium of Strategies to prevent ventilator-associated infections	✓	✓
Be able to describe interventions to prevent surgical site infections	Compare and contrast institutional policies with evidence-based practices in SHEA Compendium of Strategies to prevent surgical site infections	✓	✓
Be able to describe interventions to prevent healthcare-associated infections due to *C. difficile*	Compare and contrast institutional policies with evidence-based practices in SHEA Compendium of Strategies to prevent *C. difficile*	✓	✓
Consider the risk of transmission from contaminated and incompletely reprocessed reusable medical equipment	Perform an observation of clinical care on ≥1 unit, and propose an intervention to reduce the risk of device contamination	✓	✓
Understand the steps for reprocessing, and quality assurance of reprocessing adequacy, of devices requiring high-level disinfection and sterilization	Participate in observations of 1 or more reprocessing events: Survey the reprocessing program in the sterile processing department Observe the reprocessing of a duodenoscope and assist in performing duodenoscope cultures Conduct high-level disinfection regulatory rounds with the infection preventionist and regulatory team		✓
**Environment of care**			
Observe water safety measures including *Legionella* water monitoring	Conduct water quality surveillance with IP Participate in a water quality/safety meeting	✓ ✓	✓ ✓
Environmental cleaning—understand the evidence to support the relationship between environmental contamination and pathogen transmission	Review at least 1 published article of transmission from prior room occupant and/or environmental cultures and pathogen acquisition	✓	✓
Environmental cleaning—observe the evaluation of quality of environmental cleaning, and understand potential methods to assess environmental cleaning	Participate in environmental cleaning observations with environmental services (after discharge cleaning) or infection prevention (special case) Review the literature for evidence to support the use of fluorescent marker, adenosine triphosphate, visual inspection, and microbiologic cultures		✓ ✓
Environmental cleaning—understand the potential role for no-touch disinfection in reducing pathogen transmission	Review the BETR trial and current hospital practices for the use of no-touch disinfection		✓
Understand the methods by which Infection Prevention mitigates the risk of transmission (including pertinent pathogens involved) resulting from construction in acute care settings	Participate in observation of construction risk assessment rounds with an infection preventionist	✓	✓
Air management—be able to describe the role of airborne isolation for select pathogens including tuberculosis, and the difference in clinical and infection prevention–related risk assessments	Participate in IP team discussions related to the removal of precautions for a potential case of tuberculosis	✓	✓
Advanced air management—understand advanced principles related to air management including monitoring negative pressure, airflow in surgical settings, UV disinfection	Consider in depth at least one advanced risk and related mitigation measures in use at the facility		✓
Understand the role of life safety (environmental health and safety) in preventing infection- and non–infection-related adverse events	Identify at least 1 condition for which life safety standards may differ from standards recommended solely for infection prevention–related purposes		✓
**Quality improvement: Principles and practice**			
Understand the role of regulatory structure and oversight	Attend ≥1 multidisciplinary meeting that includes a discussion of quality improvement and accountability related to ≥1 infection-related quality measure(s)	✓	✓
Improvement tools: learn how root cause analysis and the steps of plan-do-study-act (PDSA) cycles are used to reduce risk of HAIs	Consider the application of PDSA cycles for at least 1 HAI type Observe a discussion of an HAI root cause analysis conducted by infection preventionists or quality team	✓ ✓	✓ ✓
Improvement tools: learn how advanced techniques are applied to reduce HAI PDSA, lean, six sigma Implementation science Human factor design Organizational change Failure modes and effect analysis Root cause analysis	Review educational material related to advanced techniques in quality improvement Participate in an ongoing or new quality improvement project in which 1 or more quality improvement techniques are applied		✓ ✓
Statistical methods in IP—understand the analytic methods used for quasi-experimental and observational studies	Review the SHEA research methods white papers on quasi-experimental studies and observational studies	✓	✓
Statistical methods in IP—understand the analytic methods used for randomized controlled trials, mixed methods studies, mathematical modeling	Review the SHEA research methods white papers on randomized controlled trials, mixed methods studies, and mathematical modeling Apply principles described in any of the SHEA research methods white papers to a research project		✓ ✓
Practice effective methods of education for healthcare workers and patients	Develop and administer at least 1 educational intervention in support of either a quality improvement project or HAI reduction initiative	✓	✓
**Microbiology laboratory partnership**			
Appreciate the role the microbiology laboratory plays in identification of clinical or environmental surveillance	Participate in at least 1 multidisciplinary meeting in which cultures obtained for infection prevention purposes are discussed	✓	✓
Understand the concept of diagnostic stewardship	Review ways providers’ testing choices are influenced to improve patient care related to urine cultures and *C. difficile* testing, and other diagnostic tests	✓	✓
Understand how test selection and test characteristics may impact HAI surveillance	Consider for at least 1 test the sensitivity, specificity, positive predictive value, and negative predictive value, and be able to describe the effect of these test characteristics on HAI surveillance and reporting		✓
**Antibiotic stewardship partnership**			
Appreciate the resistance profile of bacterial and fungal pathogens at the institution	Review and interpret the organizational antibiogram, and compare with rates of multidrug resistance among common HAI pathogens	✓	✓
Understand the relatedness between antibiotic use and multidrug-resistant organisms	For at least 1 common multidrug-resistant pathogen, compare HAI rates and antibiotic usage rates for pertinent antimicrobials	✓	✓
**Occupational health and infection prevention**			
Describe the impact of a healthcare worker vaccination policy on the risk of transmission of pathogens in the workplace	For at least 1 of the following pathogens, review the healthcare worker vaccination policy and consider published evidence of patient-to-HCW transmission in developed settings: influenza, hepatitis B, measles, mumps, rubella, hepatitis	✓	✓
Understand the role of presenteeism in healthcare-to-patient transmission of respiratory and gastrointestinal infections	Perform observations of healthcare worker behaviors (if rotation during respiratory virus season)		✓
(See also “Assist in an exposure investigation…” in section “Cluster detection, investigation, and resolution”)	…	…	…
**Emergency preparedness**			
Consider the role of infection prevention in prompt identification and mitigating the transmission risk of emerging pathogens	Review organizational Ebola preparedness plans	✓	✓
Review the role of emergency management and other stakeholders in preparing for seasonal epidemics	Review the organizational plans for seasonal and pandemic influenza	✓	✓
**Leadership and program implementation**			
Understand hospital administrative structure, and the internal reporting structure for infection prevention	Review the organization leadership and quality charts Attend at least 1 leadership-level meeting	✓	✓ ✓
Project management: understand the role for a project plan	Develop a project plan for the quality improvement or research project of the rotation		✓
Meeting management: understand and practice principles of successful meeting management	Review with preceptor successful strategies and barriers to efficient meetings, conduct at least one meeting, and debrief following the meeting observations of strategies employed and areas for improvement in meeting management		✓
Understand the role of a project/team charter	Review an existing infection prevention–related project/team charter, or create a new one for a fellow or team project		✓
**Supplemental and advanced topics**			
Infection prevention in non–acute-care settings			
Strategic planning			
Negotiation strategy and tactics			
Return on investment analyses			
Data visualization techniques			
Media training: print, radio, television, and web-based reporting			
Social media and nonconventional methods of professional communication			
Conference presentations (oral and poster) and manuscript writing			
Grant writing/application			
Education and mentorship of trainees			

Critically, these learning goals do not stand alone. During the IP&C/HE fellowship experience, each fellow is embedded with the IP&C team, spending dedicated time learning directly from infection preventionists, participating in observations and multidisciplinary work, and conducting analyses and quality improvement projects that promote patient safety. For fellows in fellowship training tracks (eg, antimicrobial stewardship, antibiotic resistance research, transplant ID, HIV and outpatient care, and physician-scientist), the competency activities and all other experiences are tailored to professional goals in these areas (eg, fellows interested in transplant ID may use ventricular assist device-associated infections as a case study to understand device-associated infections more broadly). This standardized, yet flexible, curriculum will help each fellow obtain a comprehensive IP&C education while allowing focused time on key areas of interest. This approach is mirrored in educational programs for other trainees, such as students pursuing a master’s degree in public health.

## Continued evolution: A living training collaborative

In this commentary, we have shared one potential framework toward formalizing the ID fellow’s training experience. Others are invited to use the competency checklist in their program (available for download at https://dom.pitt.edu/id/training/fellowshipprograms/idfellowship/programstructure/clineducpathway/). We welcome an ongoing conversation in our IP&C/HE community about the development of ID fellowship training for nascent hospital epidemiologists, including improvements upon this model, adaptations for other IP&C and healthcare epidemiology career pathways, and a medical education research agenda testing the effectiveness of this and other educational strategies.

## Supplementary material

For supplementary material accompanying this paper visit https://doi.org/10.1017/ash.2021.186. click here to view supplementary material
