# Supplementary material for: Training infectious diseases fellows for a new era of hospital epidemiology
Source: Antimicrob Steward Healthc Epidemiol. 2021 Oct 6;1(1):e25. doi: 10.1017/ash.2021.186 (PMC9495522; doi:10.1017/ash.2021.186)
Supplement: Supplementary file 1 [file S2732494X21001868sup001.docx]

| **Competency** | **Activities** | **Rotation** | **Track** |
| --- | --- | --- | --- |
| **Surveillance and Reporting** |  |  |  |
| Understand how healthcare associated infection (HAI) surveillance is conducted | Observe Infection Preventionists (IP) reviewing microbiology results and applying NHSN definitions for:   - Central line-associated bloodstream infections (CLABSI) - Catheter-associated urinary tract infections (CAUTI) - Surgical site infections (SSI) - Ventilator-associated events (VAE) - *Clostridioides (Clostridium) difficile* and multidrug-resistant organisms | ✓ | ✓ |
| Consider methods of validation and auditing of HAI data | Discuss the internal validation of HAI data  Identify the opportunities for external validation of HAI data |  | ✓  ✓ |
| Review the advantages and limitations of surveillance software in the process of HAI surveillance | Review with IP Data Analyst or Informatics team members the sources of surveillance data  Review the SHEA Research Methods white paper^1^ on administrative and surveillance data |  | ✓  ✓ |
| Distinguish between HAI surveillance and clinical definitions for infectious syndromes | Participate in a multidisciplinary discussion, quality review, or teaching session that includes case-based review describing surveillance-identified HAI | ✓ | ✓ |
| Understand and effectively utilize various HAI measures including numerator and denominator, and counts, rates, and adjusted rates | As part of routine IP work or project work, present HAI outcome data with appropriate case ascertainment, at-risk population, and consideration of risk adjustment. | ✓ | ✓ |
| Evaluate facility performance on HAI and HAI prevention process measures using internal and external benchmarks | For at least one HAI, consider and discuss internal and external benchmarking options | ✓ | ✓ |
| Consider the role of HAI in the context of other patient safety events, including falls, pressure ulcers, and other adverse outcomes | Attend ≥1 patient safety or hospital quality meeting, and compare and contrast infection-related and non-infection-related outcomes including reporting, benchmarking, and improvement interventions |  | ✓ |
| Review requirements for reporting community- and healthcare-associated infections to public health | Review state and local health Department reporting requirements  Observe IP processes for identifying reportable illnesses and performing reporting | ✓  ✓ | ✓  ✓ |
| **Cluster Detection, Investigation, and Resolution** |  |  |  |
| Describe what defines an epidemiologically significant cluster, including differences in cluster detection among pathogens | Participate in the investigation and response to a single case of an epidemiologically significant pathogen, such as vancomycin non-susceptible *Staphylococcus aureus*, carbapenem-resistant Enterobacteriaceae, *Candida auris*, *Legionella*, invasive mold, or SARS-CoV-2  (If no case is available, describe pathogens for which one or a few cases define an outbreak and those for whom clusters are differentiated from endemic rates) | ✓ | ✓ |
| For a multiple-case cluster, understand the application of multiple steps of an outbreak investigation | Participate in the investigation of a HAI cluster  (If no cluster is available, describe from the published literature at least one description of an outbreak investigation) | ✓ | ✓ |
| Understand the roles of case-control or cohort study in identifying potential transmission routes leading to a cluster of infections | Perform a case-control or cohort study as part of a cluster investigation  Describe the findings of a cluster investigation to stakeholders and hospital leadership, including proposed or enacted changes to baseline prevention practices |  | ✓  ✓ |
| Describe the role identification of genetic relatedness plays in cluster identification, and understand commonly used techniques from antimicrobial phenotype to molecular typing and whole genome sequencing (WGS) | Participate in a meeting where the use of WGS is considered for a possible cluster and/or results are reviewed  Review ≥2 published articles^2,3^ of healthcare-associated outbreaks and be able to describe the methods used to establish genetic relatedness | ✓  ✓ | ✓  ✓ |
| Understand the key elements of an exposure investigation: identifying patients and providers potentially exposed; incubation period; post-exposure measures including prophylaxis, vaccination, monitoring, furlough | Assist in an exposure investigation (e.g., tuberculosis, varicella zoster, SARS-CoV-2) in conjunction with Infection Preventionist |  | ✓ |
| Consider the role of active surveillance testing in the prevention of multidrug-resistant organisms | Compare published observational or clinical trial with institutional practice for ≥1 multidrug-resistant pathogen such as methicillin-resistant *S. aureus*, vancomycin-resistant enterococci, or carbapenem-resistant Enterobacteriaceae | ✓ | ✓ |
| **Pathogen Transmission and Transmission Interruption** |  |  |  |
| Understand modes of pathogen transmission | Be able to describe the common and potential modes of transmission of healthcare-associated pathogens | ✓ | ✓ |
| Describe the rationale for transmission-based precautions for pathogens commonly observed in the healthcare setting | Review the CDC “Type and Duration of Precautions Recommended for Selected Infections and Conditions”^4^ and compare to institutional practices | ✓ | ✓ |
| Identify clinical situations for which standard precautions (and specific elements of standard precautions) should be employed | Perform with Infection Preventionists observations of personal protective equipment use | ✓ | ✓ |
| Hand hygiene: understand the evidence base for hand hygiene as an infection prevention tool to reduce transmission in the healthcare setting | Review the World Health Organization guidelines^5^ on hand hygiene, and describe at least one high-quality study of the effectiveness of hand hygiene | ✓ | ✓ |
| Hand hygiene: observe the measurement and feedback of hand hygiene adherence | Participate in hand hygiene observations with a member of the Infection Prevention team  Conduct independent observations of hand hygiene adherence and provide feedback to observed units | ✓ | ✓  ✓ |
| Hand hygiene: understand interventions that may be employed to improve hand hygiene adherence in the acute care setting | Plan a unit-based intervention to improve hand hygiene adherence |  | ✓ |
| Be able to describe interventions to prevent catheter-related bloodstream infections | Compare and contrast institutional policies with evidence-based practices in SHEA Compendium of Strategies to prevent central line-associated bloodstream infections^6^ | ✓ | ✓ |
| Be able to describe interventions to prevent catheter-related urinary tract infections | Compare and contrast institutional policies with evidence-based practices in SHEA Compendium of Strategies to prevent catheter-associated urinary tract infections^7^ | ✓ | ✓ |
| Be able to describe interventions to prevent ventilator-associated events | Compare and contrast institutional policies with evidence-based practices in SHEA Compendium of Strategies to prevent ventilator-associated infections^8^ | ✓ | ✓ |
| Be able to describe interventions to prevent surgical site infections | Compare and contrast institutional policies with evidence-based practices in SHEA Compendium of Strategies to prevent surgical site infections^9^ | ✓ | ✓ |
| Be able to describe interventions to prevent healthcare-associated infections due to *C. difficile* | Compare and contrast institutional policies with evidence-based practices in SHEA Compendium of Strategies to prevent *C. difficile^1^*^0^ | ✓ | ✓ |
| Consider the risk of transmission from contaminated and incompletely reprocessed reusable medical equipment | Perform an observation of clinical care on ≥1 unit, and propose an intervention to reduce the risk of device contamination | ✓ | ✓ |
| Understand the steps for reprocessing, and quality assurance of reprocessing adequacy, of devices requiring high-level disinfection and sterilization | Participate in observations of one or more reprocessing events:   - Survey the reprocessing program in Sterile Processing Department - Observe the reprocessing of a duodenoscope and assist in performing duodenoscope cultures - Conduct high-level disinfection regulatory rounds with Infection Preventionist and Regulatory team |  | ✓ |
| **Environment of Care** |  |  |  |
| Observe water safety measures including Legionella water monitoring | Conduct water quality surveillance with IP  Participate in a water quality/safety meeting | ✓  ✓ | ✓  ✓ |
| Environmental cleaning – understand the evidence to support the relationship between environmental contamination and pathogen transmission | Review at least one published article of transmission from prior room occupant and/or environmental cultures and pathogen acquisition | ✓ | ✓ |
| Environmental cleaning – observe the evaluation of quality of environmental cleaning, and understand potential methods to assess environmental cleaning | Participate in environmental cleaning observations with Environmental Services (post-discharge cleaning) or Infection Prevention (special case)  Review the literature for evidence to support the use of fluorescent marker, adenosine triphosphate, visual inspection, and microbiologic cultures |  | ✓  ✓ |
| Environmental cleaning – understand the potential role for no-touch disinfection in reducing pathogen transmission | Review the BETR trial^11,12^ and current hospital practices for the use of no-touch disinfection^13^ |  | ✓ |
| Understand the methods by which Infection Prevention mitigates the risk of transmission (including pertinent pathogens involved) resulting from construction in acute care settings | Participate in observation of construction risk assessment rounds with Infection Preventionist | ✓ | ✓ |
| Air management – be able to describe the role of airborne isolation for select pathogens including tuberculosis, and the difference in clinical- and infection prevention-related risk assessments | Participate in IP team discussions related to the removal of precautions for a potential case of tuberculosis | ✓ | ✓ |
| Advanced air management – understand advanced principles related to air management including monitoring negative pressure, airflow in surgical settings, UV disinfection | Consider in depth at least one advanced risk and related mitigation measures in use at the facility |  | ✓ |
| Understand the role of Life Safety (Environmental Health and Safety) in preventing infection- and non-infection related adverse events | Identify at least one condition for which Life Safety standards may differ from standards recommended solely for Infection Prevention-related purposes |  | ✓ |
| **Quality Improvement: Principles and Practice** |  |  |  |
| Understand the role of regulatory structure and oversight | Attend ≥1 multidisciplinary meeting that includes a discussion of quality improvement and accountability related to ≥1 infection-related quality measure(s) | ✓ | ✓ |
| Improvement tools: learn how root cause analysis and the steps of Plan-Do-Study-Act (PDSA)^14^ cycles are used to reduce risk of HAIs | Consider the application of PDSA cycles for at least one HAI type  Observe a discussion of an HAI root cause analysis conducted by infection preventionists or quality team | ✓  ✓ | ✓  ✓ |
| Improvement tools: learn how advanced techniques are applied to reduce HAI^15-19^   - PDSA, Lean, Six Sigma - Implementation science - Human factor design - Organizational change - Failure modes and effect analysis - Root cause analysis | Review educational material related to advanced techniques in quality improvement  Participate in an ongoing or new quality improvement project in which one or more quality improvement techniques are applied |  | ✓  ✓ |
| Statistical methods in IP: understand the analytic methods used for quasi-experimental and observational studies | Review the SHEA Research Methods white papers on quasi-experimental studies^20^ and observational studies^21^ | ✓ | ✓ |
| Statistical methods in IP: understand the analytic methods used for randomized controlled trials, mixed methods studies, mathematical modeling | Review the SHEA Research Methods white papers on randomized controlled trials,^22^ mixed methods studies, and mathematical modeling^23^  Apply principles described in any of the SHEA Research Methods white papers^24^ to a research project |  | ✓  ✓ |
| Practice effective methods of education for healthcare workers and patients | Develop and administer at least one educational intervention in support of either a quality improvement project or HAI reduction initiative | ✓ | ✓ |
| **Microbiology Laboratory Partnership** |  |  |  |
| Appreciate the role the Microbiology Laboratory plays in identification of clinical or environmental surveillance | Participate in at least one multidisciplinary meeting in which cultures obtained for Infection Prevention purposes are discussed | ✓ | ✓ |
| Understand the concept of diagnostic stewardship^25^ | Review ways providers’ testing choices are influenced to improve patient care related to urine cultures and *C. difficile* testing, and other diagnostic tests | ✓ | ✓ |
| Understand how test selection and test characteristics may impact HAI surveillance | Consider for at least one test the sensitivity, specificity, positive predictive value, and negative predictive value, and be able to describe the effect of these test characteristics on HAI surveillance and reporting |  | ✓ |
| **Antibiotic Stewardship Partnership** |  |  |  |
| Appreciate the resistance profile of bacterial and fungal pathogens at the institution | Review and interpret the organizational antibiogram, and compare with rates of multidrug-resistance among common HAI pathogens | ✓ | ✓ |
| Understand the relatedness between antibiotic use and multidrug-resistant organisms | For at least one common multidrug-resistant pathogen, compare HAI rates and antibiotic usage rates for pertinent antimicrobials. | ✓ | ✓ |
| **Occupational Health and Infection Prevention** |  |  |  |
| Describe the impact of a healthcare worker vaccination policy on the risk of transmission of pathogens in the workplace | For at least one of the following pathogens, review the healthcare worker vaccination policy and consider published evidence of patient-to-healthcare worker transmission in developed settings: influenza, hepatitis B, measles, mumps, rubella, hepatitis | ✓ | ✓ |
| Understand the role of presenteeism in healthcare-to-patient transmission of respiratory and gastrointestinal infections^26^ | Perform observations of healthcare worker behaviors (if rotation during respiratory virus season) |  | ✓ |
| (See also “Assist in an exposure investigation...” in section “Cluster Detection, Investigation, and Resolution”) | — | — | — |
| **Emergency Preparedness** |  |  |  |
| Consider the role of Infection Prevention in prompt identification and mitigating the transmission risk of emerging pathogens | Review organizational Ebola preparedness plans | ✓ | ✓ |
| Review the role of Emergency Management and other stakeholders in preparing for seasonal epidemics | Review the organizational plans for seasonal and pandemic influenza | ✓ | ✓ |
| **Leadership and Program Implementation** |  |  |  |
| Understand hospital administrative structure, and the internal reporting structure for Infection Prevention | Review the organization leadership and quality charts  Attend at least one leadership-level meeting | ✓ | ✓  ✓ |
| Project management: understand the role for a project plan | Develop a project plan for the quality improvement or research project of the rotation |  | ✓ |
| Meeting management: understand and practice principles of successful meeting management | Review with preceptor successful strategies and barriers to efficient meetings, conduct at least one meeting, and debrief following the meeting observations of strategies employed and areas for improvement in meeting management |  | ✓ |
| Understand the role of a project/team charter | Review an existing Infection Prevention-related project/team charter, or create a new one for a fellow or team project |  | ✓ |
| **Supplemental and Advanced Topics** |  |  |  |
| Infection Prevention in non-acute care settings |  |  |  |
| Strategic planning |  |  |  |
| Negotiation strategy and tactics |  |  |  |
| Return on investment analyses |  |  |  |
| Data visualization techniques |  |  |  |
| Media training: print, radio, television, and web-based reporting |  |  |  |
| Social media and non-conventional methods of professional communication |  |  |  |
| Conference presentations (oral and poster) and manuscript writing |  |  |  |
| Grant writing/application |  |  |  |
| Education and mentorship of trainees |  |  |  |

**REFERENCES**

**1.** Drees M, Gerber JS, Morgan DJ, Lee GM. Research Methods in Healthcare Epidemiology and Antimicrobial Stewardship: Use of Administrative and Surveillance Databases. *Infect Control Hosp Epidemiol* 2016;37:1278-1287.

**2.** Sundermann AJ, Babiker A, Marsh JW, et al. Outbreak of Vancomycin-resistant Enterococcus faecium in Interventional Radiology: Detection Through Whole-genome Sequencing-based Surveillance. *Clin Infect Dis* 2020;70:2336-2343.

**3.** Sundermann AJ, Chen J, Miller JK, et al. Outbreak of Pseudomonas aeruginosa Infections from a Contaminated Gastroscope Detected by Whole Genome Sequencing Surveillance. *Clin Infect Dis* 2020.

**4.** Siegel JD, Rhinehart E, Jackson M, Chiarello L, Health Care Infection Control Practices Advisory C. 2007 Guideline for Isolation Precautions: Preventing Transmission of Infectious Agents in Health Care Settings. *Am J Infect Control* 2007;35:S65-164.

**5.** Sax H, Allegranzi B, Chraiti MN, Boyce J, Larson E, Pittet D. The World Health Organization hand hygiene observation method. *Am J Infect Control* 2009;37:827-834.

**6.** Marschall J, Mermel LA, Fakih M, et al. Strategies to prevent central line-associated bloodstream infections in acute care hospitals: 2014 update. *Infect Control Hosp Epidemiol* 2014;35 Suppl 2:S89-107.

**7.** Lo E, Nicolle LE, Coffin SE, et al. Strategies to prevent catheter-associated urinary tract infections in acute care hospitals: 2014 update. *Infect Control Hosp Epidemiol* 2014;35 Suppl 2:S32-47.

**8.** Klompas M, Branson R, Eichenwald EC, et al. Strategies to prevent ventilator-associated pneumonia in acute care hospitals: 2014 update. *Infect Control Hosp Epidemiol* 2014;35 Suppl 2:S133-154.

**9.** Anderson DJ, Podgorny K, Berrios-Torres SI, et al. Strategies to prevent surgical site infections in acute care hospitals: 2014 update. *Infect Control Hosp Epidemiol* 2014;35 Suppl 2:S66-88.

**10.** Dubberke ER, Carling P, Carrico R, et al. Strategies to prevent Clostridium difficile infections in acute care hospitals: 2014 update. *Infect Control Hosp Epidemiol* 2014;35 Suppl 2:S48-65.

**11.** Anderson DJ, Chen LF, Weber DJ, et al. Enhanced terminal room disinfection and acquisition and infection caused by multidrug-resistant organisms and Clostridium difficile (the Benefits of Enhanced Terminal Room Disinfection study): a cluster-randomised, multicentre, crossover study. *Lancet* 2017;389:805-814.

**12.** Anderson DJ, Moehring RW, Weber DJ, et al. Effectiveness of targeted enhanced terminal room disinfection on hospital-wide acquisition and infection with multidrug-resistant organisms and Clostridium difficile: a secondary analysis of a multicentre cluster randomised controlled trial with crossover design (BETR Disinfection). *Lancet Infect Dis* 2018;18:845-853.

**13.** Han JH, Sullivan N, Leas BF, Pegues DA, Kaczmarek JL, Umscheid CA. Cleaning Hospital Room Surfaces to Prevent Health Care-Associated Infections: A Technical Brief. *Ann Intern Med* 2015;163:598-607.

**14.** Langley GJ. *The improvement guide : a practical approach to enhancing organizational performance.* 2nd ed. San Francisco: Jossey-Bass; 2009.

**15.** Sreeramoju PV, Palmore TN, Lee GM, et al. Institutional quality and patient safety programs: An overview for the healthcare epidemiologist. *Infect Control Hosp Epidemiol* 2021;42:6-17.

**16.** Liker JK, Meier D. *The Toyota way fieldbook : a practical guide for implementing Toyota's 4Ps.* New York: McGraw-Hill; 2006.

**17.** DelliFraine JL, Langabeer JR, 2nd, Nembhard IM. Assessing the evidence of Six Sigma and Lean in the health care industry. *Qual Manag Health Care* 2010;19:211-225.

**18.** Harry MJ, Schroeder R. *Six sigma : the breakthrough management strategy revolutionizing the world's top corporations.* 1st ed. New York: Currency; 2000.

**19.** Saint S, Howell JD, Krein SL. Implementation science: how to jump-start infection prevention. *Infect Control Hosp Epidemiol* 2010;31 Suppl 1:S14-17.

**20.** Schweizer ML, Braun BI, Milstone AM. Research Methods in Healthcare Epidemiology and Antimicrobial Stewardship-Quasi-Experimental Designs. *Infect Control Hosp Epidemiol* 2016;37:1135-1140.

**21.** Snyder GM, Young H, Varman M, Milstone AM, Harris AD, Munoz-Price S. Research Methods in Healthcare Epidemiology and Antimicrobial Stewardship-Observational Studies. *Infect Control Hosp Epidemiol* 2016;37:1141-1146.

**22.** Anderson DJ, Juthani-Mehta M, Morgan DJ. Research Methods in Healthcare Epidemiology and Antimicrobial Stewardship: Randomized Controlled Trials. *Infect Control Hosp Epidemiol* 2016;37:629-634.

**23.** Barnes SL, Kasaie P, Anderson DJ, Rubin M. Research Methods in Healthcare Epidemiology and Antimicrobial Stewardship-Mathematical Modeling. *Infect Control Hosp Epidemiol* 2016;37:1265-1271.

**24.** Morgan DJ, Safdar N, Milstone AM, Anderson DJ. Research Methods in Healthcare Epidemiology and Antimicrobial Stewardship. *Infect Control Hosp Epidemiol* 2016;37:627-628.

**25.** Madden GR, Weinstein RA, Sifri CD. Diagnostic Stewardship for Healthcare-Associated Infections: Opportunities and Challenges to Safely Reduce Test Use. *Infect Control Hosp Epidemiol* 2018;39:214-218.

**26.** Szymczak JE, Smathers S, Hoegg C, Klieger S, Coffin SE, Sammons JS. Reasons Why Physicians and Advanced Practice Clinicians Work While Sick: A Mixed-Methods Analysis. *JAMA Pediatr* 2015;169:815-821.
